# Supplementary material for: Functional heterogeneity of lymphocytic patterns in primary melanoma dissected through single-cell multiplexing
Source: eLife. 2020 Feb 14;9:e53008. doi: 10.7554/eLife.53008 (PMC7053517; doi:10.7554/eLife.53008)
Supplement: Transparent reporting form [file elife-53008-transrepform.docx]

***eLife’s* transparent reporting form**

We encourage authors to provide detailed information *within their submission* to facilitate the interpretation and replication of experiments. Authors can upload supporting documentation to indicate the use of appropriate reporting guidelines for health-related research (see [EQUATOR Network](http://www.equator-network.org/%20)), life science research (see the [BioSharing Information Resource](https://biosharing.org/)), or the [ARRIVE guidelines](http://www.plosbiology.org/article/info:doi/10.1371/journal.pbio.1000412) for reporting work involving animal research. Where applicable, authors should refer to any relevant reporting standards documents in this form.

If you have any questions, please consult our Journal Policies and/or contact us: [editorial@elifesciences.org](mailto:editorial@elifesciences.org).

**Sample-size estimation**

- You should state whether an appropriate sample size was computed when the study was being designed
- You should state the statistical method of sample size computation and any required assumptions
- If no explicit power analysis was used, you should describe how you decided what sample (replicate) size (number) to use

Please outline where this information can be found within the submission (e.g., sections or figure legends), or explain why this information doesn’t apply to your submission:

Our single-cell study of the melanoma microenvironment involves the proteomic profile and spatial distribution of 242224 cells measured in 60 TMA cores, corresponding to a total of 29 patients. With these massive numbers, classic statistical tests that depend on sample size would report misleading results (the statistical approach followed in the present study is discussed below).

At the current time, this number of patients (29) is at the upper end of common practice and of golden standard in the field of single-cell proteomics. Lim. et al. (Gut 2019) examined 23 patients with hepatocellular carcinoma and Cader et al. (Blood 2018) included 7 patients with classical Hodgkin lymphoma and 10 controls in similar studies of the tumor microenvironment.

**Replicates**

- You should report how often each experiment was performed
- You should include a definition of biological versus technical replication
- The data obtained should be provided and sufficient information should be provided to indicate the number of independent biological and/or technical replicates
- If you encountered any outliers, you should describe how these were handled
- Criteria for exclusion/inclusion of data should be clearly stated
- High-throughput sequence data should be uploaded before submission, with a private link for reviewers provided (these are available from both GEO and ArrayExpress)

Please outline where this information can be found within the submission (e.g., sections or figure legends), or explain why this information doesn’t apply to your submission:

Due to the nature of the experimental approach followed in this study, technical replicates were impossible to obtain.

Biological tissue is highly heterogeneous. Thus, whenever possible (i.e. when the melanoma was big enough), biological replicates were obtained (between 1 and 5 cores from each patient).

To minimize the effect of potential outliers in terms of protein expression, each marker was z-normalized and their values trimmed in the [-5, 5] range (materials and methods, image pre-processing, page 8, lines 7 to 21).

For the cells, the following inclusion criteria was followed: those cells that did not have expression in at least 3 protein markers were removed. Moreover, those proteins that were not expressed in at least 1% of the cells were removed. All the cells passed the inclusion criteria described above (materials and methods, image pre-processing, page 8, lines 7 to 21).

For the cell clusters, the following inclusion criteria was followed: those cells who agreed in their cell type assignation in at least two out of the three clustering methods were included. Those cells in which the three clustering methods assigned different cell phenotypes were excluded (Materials and method, phenotypic identification, page 9, lines 4 to 23 and supplementary data figure 2). Moreover, those cells that could not be mapped to known cell types were removed. In total, 62920 from 242224 cells were removed (25.98%) (results, page 4, lines 11 to 15).

**Statistical reporting**

- Statistical analysis methods should be described and justified
- Raw data should be presented in figures whenever informative to do so (typically when N per group is less than 10)
- For each experiment, you should identify the statistical tests used, exact values of N, definitions of center, methods of multiple test correction, and dispersion and precision measures (e.g., mean, median, SD, SEM, confidence intervals; and, for the major substantive results, a measure of effect size (e.g., Pearson's r, Cohen's d)
- Report exact p-values wherever possible alongside the summary statistics and 95% confidence intervals. These should be reported for all key questions and not only when the p-value is less than 0.05.

Please outline where this information can be found within the submission (e.g., sections or figure legends), or explain why this information doesn’t apply to your submission:

With these massive numbers, classic statistical tests that depend on sample size would report misleading results. The specific statistical methods used are described in the main manuscript (Materials and methods: from page 8, line 7; to page 10, line 3).

(For large datasets, or papers with a very large number of statistical tests, you may upload a single table file with tests, Ns, etc., with reference to sections in the manuscript.)

**Group allocation**

- Indicate how samples were allocated into experimental groups (in the case of clinical studies, please specify allocation to treatment method); if randomization was used, please also state if restricted randomization was applied
- Indicate if masking was used during group allocation, data collection and/or data analysis

Please outline where this information can be found within the submission (e.g., sections or figure legends), or explain why this information doesn’t apply to your submission:

Patients were assigned to the brisk and nonbrisk categories after revision of the slides by two expert dermatopathologists (FMB, JVDO). Patients were assigned into active/exhausted groups based on the analysis described in materials and methods, functional analysis of TILs, page 8, lines 23 to 45.

During unsupervised data analysis, sample labels were masked to avoid methodological biases.

**Additional data files (“source data”)**

- We encourage you to upload relevant additional data files, such as numerical data that are represented as a graph in a figure, or as a summary table
- Where provided, these should be in the most useful format, and they can be uploaded as “Source data” files linked to a main figure or table
- Include model definition files including the full list of parameters used
- Include code used for data analysis (e.g., R, MatLab)
- Avoid stating that data files are “available upon request”

Please indicate the figures or tables for which source data files have been provided:

We tried to upload the data files but we encountered the following error message: “only files under 100Mb can be uploaded”. Single-cell multiplexing data are too large to be easily shared. The codes used for this manuscript are shared compressed in a zip file under the name Code.zip.
